# Late age at first birth is a protective factor for oesophageal cancer and gastro-oesophageal reflux: the evidence from the genetic study

**DOI:** 10.3389/fendo.2023.1329763

**Published:** 2024-01-15

**Authors:** Yani Su, Yiwei Xu, Yunfeng Hu, Yu Chang, Fangcai Wu, Mingyi Yang, Yuhui Peng

**Affiliations:** ^1^ Department of Clinical Laboratory Medicine, Cancer Hospital of Shantou University Medical College, Shantou, China; ^2^ Department of Radiotherapy, Yan’an University Affiliated Hospital, Yan’an, China; ^3^ Department of Radiation Oncology, Cancer Hospital of Shantou University Medical College, Shantou, China; ^4^ Department of Joint Surgery, HongHui Hospital, Xi’an Jiaotong University, Xi’an, Shaanxi, China

**Keywords:** age at first birth, oesophageal cancer, gastro-oesophageal reflux, genetic, causal

## Abstract

**Objective:**

The primary objective of this research endeavor was to examine the underlying genetic causality between the age at first birth (AFB) and four prevalent esophageal diseases, namely oesophageal obstruction (OO), oesophageal varices (OV), gastro-oesophageal reflux (GOR), and oesophageal cancer (OC).

**Methods:**

We conducted a two-sample Mendelian randomization (MR) analysis to examine the causal association between AFB and four prevalent esophageal disorders. We employed eight distinct MR analysis techniques to evaluate causal relationships, encompassing random-effects inverse variance weighted (IVW), MR Egger, weighted median, simple mode, weighted mode, maximum likelihood, penalized weighted median, and fixed-effects IVW. The random-effects IVW method served as the primary approach for our analysis. Furthermore, we executed several sensitivity analyses to assess the robustness of the genetic causal inferences.

**Results:**

The random-effects IVW analysis revealed a significant negative genetic causal association between AFB and both GOR (P < 0.001, Odds Ratio [OR] 95% Confidence Interval [CI] = 0.882 [0.828-0.940]) and OC (P < 0.001, OR 95% CI = 0.998 [0.998-0.999]). Conversely, there was insufficient evidence support to substantiate a genetic causal link between AFB and OO (P = 0.399, OR 95% CI = 0.873 [0.637-1.197]) or OV (P = 0.881, OR 95% CI = 0.978 [0.727-1.314]). The results of sensitivity analyses underscore the robustness and reliability of our MR analysis.

**Conclusion:**

The findings of this investigation substantiate the notion that elevated AFB confers a protective effect against GOR and OC. In addition, no causative association was discerned between AFB and OO or OV at the genetic level.

## 1 Introduction

Oesophageal cancer (OC) constitutes a pressing global health concern. In 2012, an estimated 456,000 cases of OC were diagnosed worldwide (1). The prognosis for OC patients is bleak, with a five-year survival rate of less than 20% observed across all patients, even in developed nations such as the United States (2). Given the advanced stage at which most OC cases are detected and their associated high mortality rates, early identification represents a pivotal means of enhancing patient outcomes (3). Recognized risk factors for OC include smoking, alcohol consumption, and obesity (4). OC stands as the eighth most prevalent cancer globally and ranks as the sixth leading cause of cancer-related mortality worldwide. This alarming statistic has prompted increased awareness among both the general public and healthcare professionals, emphasizing the imperative need to mitigate OC-related fatalities (3). The two principal approaches to curbing OC mortality are primary prevention, involving the regulation of smoking and alcohol consumption, adoption of a healthful diet, and management of obesity, and the timely identification and treatment of the disease. Early detection strategies, in conjunction with interventions targeting precancerous lesions, have the potential to ameliorate OC outcomes. Gastroesophageal reflux (GOR) has garnered recognition as a precancerous lesion associated with OC. GOR represents a prevalent affliction affecting both adults and children, with its global prevalence on the rise. The pathophysiology of GOR is intricate, involving various contributing mechanisms that yield GOR symptoms. These mechanisms encompass factors such as gastric composition, motility, anti-reflux barrier function, refluxate characteristics, clearance mechanisms, mucosal integrity, and symptom perception (5). Notably, the dilatation of the muscular esophageal wall can obscure the obstructive effect of intrinsic or exogenous tumors, often evading detection until the disease has penetrated deep into the muscular layers and extended to involve lymph nodes and beyond (3). Consequently, to more effectively prevent OC incidence and enhance early detection, it is imperative to afford attention to certain clinically prevalent esophageal conditions, including esophageal obstruction (OO) and esophageal varices (OV).

OC, a malignancy that is both inadequately investigated and exceedingly lethal, exhibits a pronounced gender disparity, wherein men are afflicted with OC at a rate three to four times higher than that of women (6). This gender dichotomy in OC incidence is intricately associated with the dissimilarities in adipose tissue distribution between the sexes. Specifically, men tend to accumulate greater amounts of visceral adipose tissue, while women predominantly store subcutaneous fat deposits (7). Sex hormones wield a pivotal influence over body fat distribution. Estrogen, a chief female sex hormone, fosters the deposition of subcutaneous fat as opposed to visceral adiposity, and a decline in estrogen levels, particularly among postmenopausal women, is correlated with an augmented presence of visceral fat reserves (8). Furthermore, the influence of sex hormones extends beyond their role in fat distribution, as they are intimately connected to the substantial male gender bias observed in OC incidence. Disparities in estrogen expression or its associated signaling pathways may underpin this gender skew (9). Estrogen, being a principal female sex hormone, is intricately linked mechanistically to various facets of cancer susceptibility and cancer development (9). Consequently, it is reasonable to think that estrogen may serve as a contributory factor to the gender discrepancy in OC incidence. Empirical research has shown that sex hormones, particularly estrogen, possess the capacity to mitigate the onset of OC (10, 11). Fundamental investigations have demonstrated that estrogen exerts an inhibitory influence on the proliferation of OC cells, with estrogen receptors likely mediating this protective mechanism (12). Estrogen receptor subtypes α and β hold prognostic significance in OC patients (13). Notably, estrogen-associated receptor α has been identified as an instigator of mitochondrial biogenesis in OC and a factor conferring resistance to neoadjuvant chemoradiotherapy (14). In addition to its role in OC pathogenesis, estrogen plays a pivotal role in the context of GOR. Research indicates that estrogen can engender deleterious effects in the context of GOR, but it can also be leveraged to shield the mucosa from GOR-induced damage and its ensuing complications, including metaplasia and malignancy (15). The utilization of estrogen in the management of erosive reflux and the prevention of associated complications represents a potentially promising avenue for future research. A wealth of evidence underscores the pivotal role of estrogen in both OC and GOR, thereby motivating investigations into the interplay between OC, GOR, and reproductive factors such as age at first birth (AFB), age at menarche, and age at menopause.

Mendelian randomization (MR) is a kind of data analysis method which is mainly used in epidemiological etiological inference in recent years. Different genotypes determine different intermediate phenotypes. If the phenotype represents a certain exposure characteristic of an individual, the association effect between genotype and disease can represent the effect of exposure factors on disease. Since alleles follow the principle of random allocation, the effect is not affected by confounding factors and reverse causation in traditional epidemiological studies (16). Reverse causation, where the chronological order of exposure and outcome is reversed. MR represents a statistical methodology employed to investigate the genetic underpinnings of causality within the context of exposure and its associated outcomes. In the pursuit of establishing a causal nexus between exposure and outcomes, MR leverages single nucleotide polymorphisms (SNPs) that meet three fundamental assumptions and displaying substantial correlations with the exposure in question, as instrumental variables (IVs), effectively serving as proxy variables for the exposure (17). The robustness and potential of MR in unraveling the causal relationships between exposures and their respective outcomes have been well-demonstrated in prior research. For example, prior investigations have employed MR to explore the causal links between age at menarche, age at menopause, and the development of oesophageal neoplasia (18). Furthermore, MR has been utilized to scrutinize the causal association between AFB and the lung cancer (19). In the present study, our primary objective is to employ MR analysis to investigate the genetic causality between AFB and four prevalent esophageal disorders, specifically, OO, OV, GOR, and OC.

## 2 Materials and methods

### 2.1 Study design

**Figure 1:**
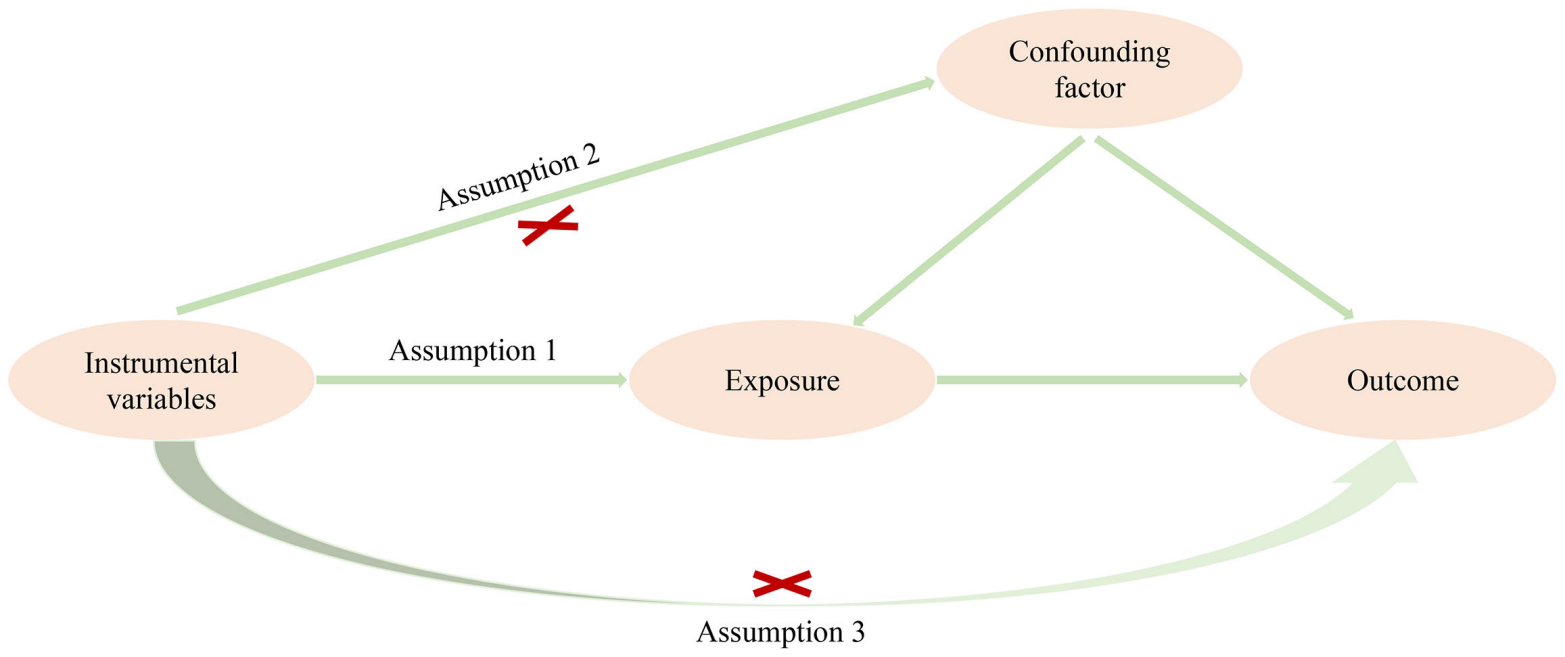
Three basic assumptions of MR analysis.

In this study, we conducted a two-sample MR analysis, utilizing AFB as the exposure variable and investigating its potential influence on four distinct esophageal diseases, namely, OO, OV, GOR, and OC. The MR analysis adhered rigorously to the fundamental assumptions of this method, which are as follows: (1) the IVs exhibit a strong correlation with the exposure of interest, in this case, AFB; (2) the IVs do not exhibit associations with the outcomes under study or any confounding factors; and (3) the IVs can solely affect the outcomes through exposure variable, as detailed in **Figure 1**. The genetic information used in this investigation was derived from publicly accessible genome-wide association study (GWAS) summary data, obviating the necessity for obtaining informed patient consent and ethical statements for the execution of this research.

### 2.2 Data source

The summary data for GWAS of exposure and outcomes were obtained from the IEU OpenGWAS database, which can be accessed at the following URL: https://gwas.mrcieu.ac.uk/. The GWAS summary data for AFB comprised a dataset of 542,901 samples and 9,702,772 SNPs. The summary data for OO, OV, and GOR were generated by the Finnish consortium. The dataset for OO included 190,156 samples and 16,380,373 SNPs, while the dataset for OV encompassed 190,513 samples and 16,380,395 SNPs. The GOR dataset consisted of 202,836 samples and 16,380,425 SNPs. All case subjects were identified based on the application of the M13 code in the International Classification of Diseases-Tenth Revision (ICD-10). Genotyping procedures were conducted using Illumina technology (Illumina Inc, San Diego) and Affymetrix chip arrays (Thermo Fisher Scientific, Santa Clara, CA, USA). For more comprehensive insights into the data employed, interested parties are encouraged to visit the FinnGen website. The GWAS summary data for OC were generated by the UK Biobank, featuring a dataset with 372,756 samples and 8,970,465 SNPs. It is important to note that all participants in this study, both exposure and outcomes, were of European descent. For a detailed breakdown of the data utilized in this analysis, please refer to **Supplementary Table 1**.

### 2.3 IVs selection

The rigorous quality control of IVs plays a pivotal role in ensuring the reliability of MR analysis. The selection of IVs must adhere strictly to the three fundamental assumptions that underlie MR analysis. Firstly, SNPs employed as IVs should exhibit a robust correlation with the exposure of interest (here denoted as AFB). The criteria for establishing this strong correlation are defined as having a P-value < 5 x 10^-8 and an F statistic > 10. The F statistic is computed using the formula: F = R^2(N-K-1)/K(1-R^2). Secondly, a meticulous screening process is implemented to mitigate the influence of linkage disequilibrium (LD), with only those SNPs demonstrating minimal LD (LD r^2 < 0.001 and a clump distance > 10,000 kb) retained as IVs. Thirdly, the selected SNPs utilized as IVs should not exhibit any correlation with the outcome variable of interest. The criteria for establishing this lack of correlation are set at a P-value < 5 x 10^-8. Fourthly, the chosen SNPs as IVs must not be associated with potential confounding factors. To further control for confounding variables, the PhenoScanner database is employed, facilitating the mitigation of the impact of extraneous covariates. In the present study, the identified confounding factors for the respective outcomes are as follows: for “OO,” the confounding factor is esophageal atresia (20); for “OV,” diabetes (21) is a confounding factor; “GOR” is affected by old age and body mass index (22); and “OC” is influenced by smoking, alcohol consumption, and obesity (23, 24). Fifthly, SNPs characterized by palindromic sequences and intermediate allele frequencies are systematically excluded from the analytical process (25).

### 2.4 MR analysis

In alignment with standard practice in MR analyses, the primary analytical approach employed in this investigation is the random-effects inverse variance weighted (IVW) method (26, 27). This study also incorporates a spectrum of other MR methodologies to assess the causal associations between exposure and outcome, encompassing MR Egger, weighted median, simple mode, and weighted mode. Furthermore, three MR analytical approaches, namely maximum likelihood, penalized weighted median, and fixed-effects IVW, were utilized to corroborate the findings derived from the random-effects IVW analysis. The preeminence of the random-effects IVW method in MR analysis stems from its robust statistical properties, which engender a heightened reliance on its analytical outcomes. The random-effects IVW method, predicated upon the amalgamation of Wald estimates corresponding to each IVs, imparts a coherent capability for the evaluation of genetic causality between exposure and outcome (28). In the absence of horizontal pleiotropy, the random-effects IVW method demonstrates an intrinsic capacity to deliver a relatively uniform and precise assessment of genetic causation.

### 2.5 Sensitivity analysis

In order to ascertain the robustness and credibility of genetic causal inferences pertaining to exposure and outcomes, a comprehensive set of sensitivity analyses was undertaken to corroborate the MR analysis results previously expounded. Firstly, we employed two distinct techniques to assess heterogeneity within the MR analyses, encompassing the utilization of Cochran’s Q statistic in the context of MR-IVW, and the application of Rucker’s Q statistic in conjunction with MR Egger. To address concerns of horizontal pleiotropy, we implemented a duo of statistical tests, namely the MR Egger intercept test and the MR Pleiotropy Residual Sum and Outlier (MR-PRESSO) methodology. Notably, the MR-PRESSO methodology was also employed to identify potential outliers in the MR analysis. In order to investigate the influence of individual SNPs on genetic causal assessments of exposure and outcomes, a “Leave-one-out” analysis was introduced. Finally, the MR Robust Adjusted Profile Score (MR-RAPS) method was employed to assess the assumption of normality in the MR analysis.

### 2.6 Statistical analysis

The analysis encompassed the application of the “TwoSampleMR” software package for the purpose of conducting two-sample MR investigations. Additionally, the “MRPRESSO” software package was utilized to perform the MR-PRESSO test. These analytical procedures were executed within the framework of R version 4.1.2. The significance threshold was established at P-value < 0.05 to make inferences regarding genetic causation. Specifically, a P-value < 0.05 coupled with an Odds Ratio (OR) > 1 signified a positive genetic causal relationship, while an OR < 1 denoted the presence of a negative genetic causal association. Moreover, instances where the P-value > 0.05 were indicative of the absence of heterogeneity, horizontal pleiotropy, and adherence to the assumptions of a normal distribution.

## 3 Results

### 3.1 IVs selection

We identified a total of 67 SNPs that exhibited strong correlations with AFB. These 67 SNPs are present within the GWAS summary data pertaining to three distinct outcomes: OO, OV, and GOR. It is noteworthy that none of these 67 SNPs demonstrated significant associations with any of the three aforementioned outcomes (OO, OV, GOR) or their respective confounding variables. However, among these SNPs, 12 were identified as palindrome SNPs (rs10445366, rs10752613, rs13319205, rs13420733, rs13420733, rs1464534, rs1590949, rs2530597, rs4443016, rs5763436, rs62261746, rs7958796). As a result of excluding the palindrome SNPs from the analysis, we retained 55 IVs that were suitable for genetic causal assessment of AFB and OO, OV, or GOR, as detailed in **Supplementary Tables 2, 3**, and **4**. During the MR analysis concerning the relationship between AFB and OC, a total of 65 SNPs were identified within the GWAS summary data for OC. Notably, 11 of these SNPs were found to be associated with confounding variables (rs11081529, rs13319205, rs1464534, rs1702877, rs17314804, rs17391694, rs1859100, rs2530597, rs55988458, rs590076, rs62261746), and 11 SNPs exhibited palindromic characteristics (rs10445366, rs10752613, rs13319205, rs13420733, rs1464534, rs1590949, rs2530597, rs4443016, rs5763436, rs62261746, rs7958796). It is worth noting that four SNPs (rs2530597, rs13319205, rs62261746, rs1464534) were identified as both confounding-related SNPs and palindrome SNPs. Following the removal of SNPs associated with confounding factors and palindrome SNPs, a total of 47 IVs were retained for the purpose of assessing genetic causality in the relationship between AFB and OC, as documented in **Supplementary Table 5**.

### 3.2 MR analysis and sensitivity analysis

**Figure 2:**
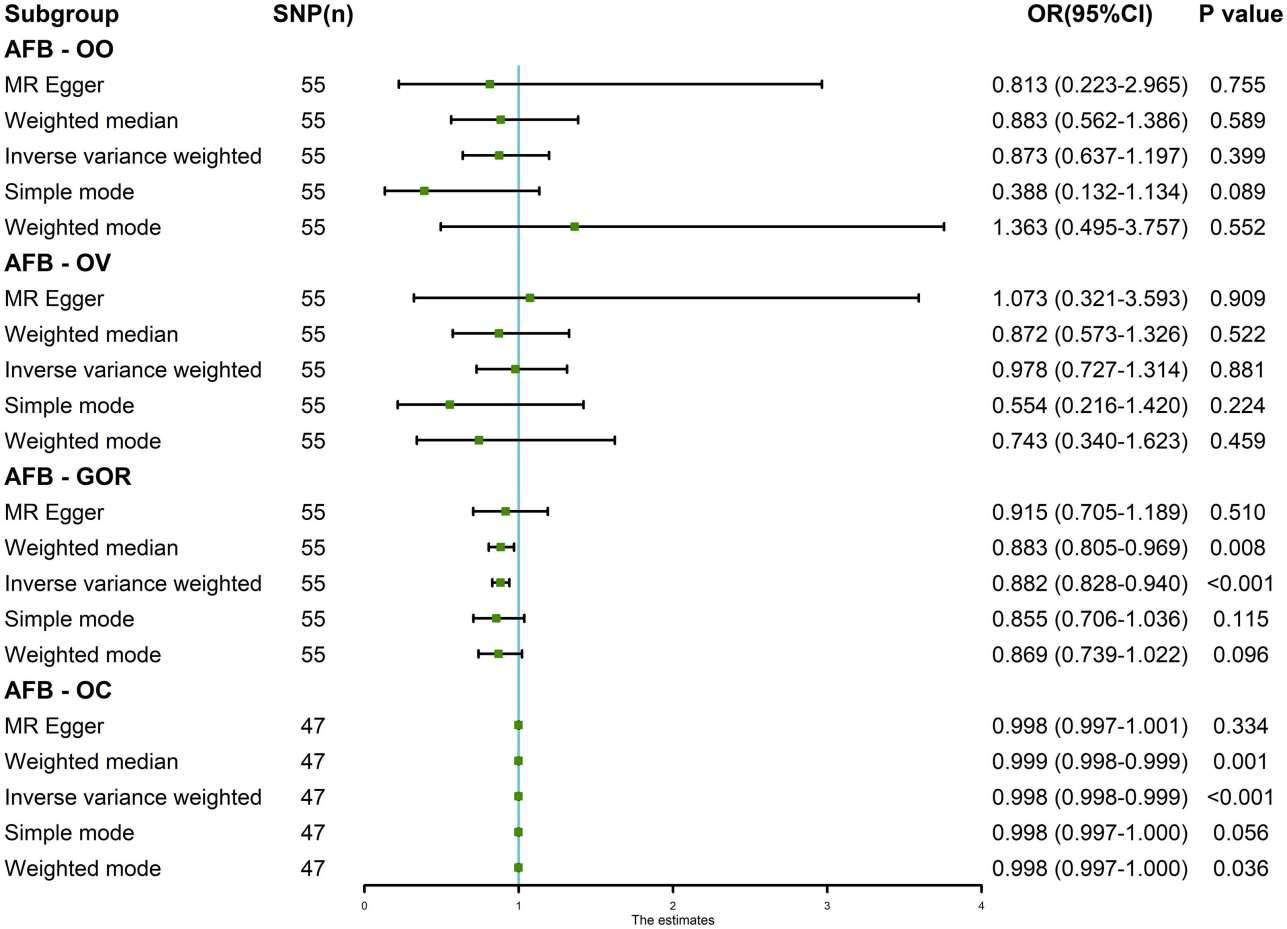
The MR analysis results of age at first birth and four common esophageal diseases, including oesophageal obstruction, oesophageal varices, gastro-oesophageal reflux and oesophageal cancer. The analysis employed five methods, namely random-effects IVW, MR Egger, weighted median, simple mode and weighted mode.

**Figure 3:**
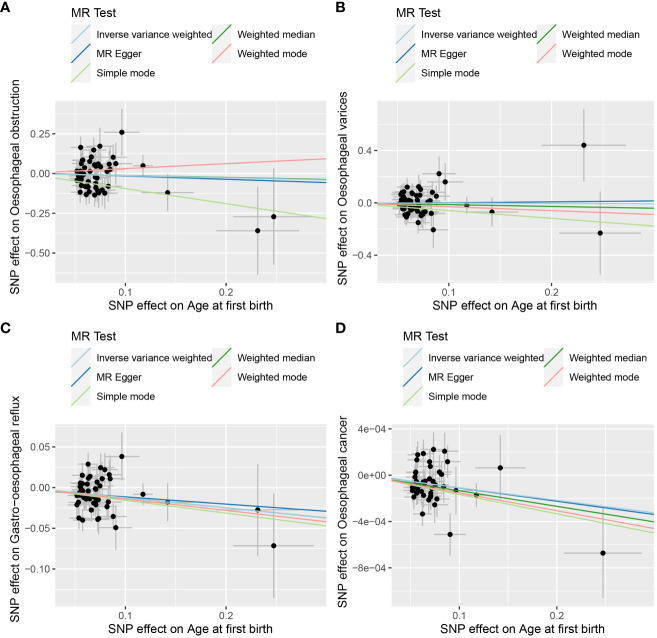
The scatter plot of MR analysis between age at first birth and four common esophageal diseases. **(A)** age at first birth and oesophageal obstruction; **(B)** age at first birth and oesophageal varices; **(C)** age at first birth and gastro-oesophageal reflux; **(D)** age at first birth and oesophageal cancer.

The results of the random-effects IVW analysis revealed no discernible genetic causal association between AFB and either OO (P = 0.399, OR 95% Confidence Interval [CI] = 0.873 [0.637-1.197]) or OV (P = 0.881, OR 95% CI = 0.978 [0.727-1.314]). Various alternative analytical approaches, including MR Egger, the weighted median, the simple mode, and the weighted mode, all substantiate the findings of the random-effects IVW analysis (P > 0.05). Furthermore, the random-effects IVW analysis unveiled a notable negative genetic causal relationship between AFB and both GOR (P < 0.001, OR 95% CI = 0.882 [0.828-0.940]) and OC (P < 0.001, OR 95% CI = 0.998 [0.998-0.999]). Of the alternative analytical methods, only the weighted median approach supports the presence of a negative genetic causal link between AFB and GOR (P < 0.05), while both the weighted median and weighted mode methods substantiate the existence of a negative genetic causal relationship between AFB and OC (P < 0.05), as detailed in **Figures 2**, **3**.

**Figure 4:**
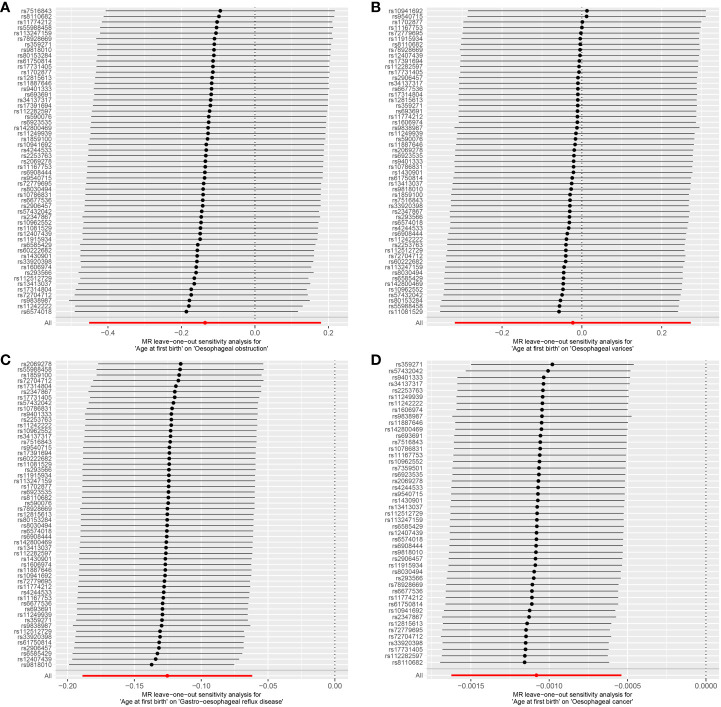
The leave-one-out analysis between age at first birth and four common esophageal diseases. **(A)** age at first birth and oesophageal obstruction; **(B)** age at first birth and oesophageal varices; **(C)** age at first birth and gastro-oesophageal reflux; **(D)** age at first birth and oesophageal cancer.

**Figure 5:**
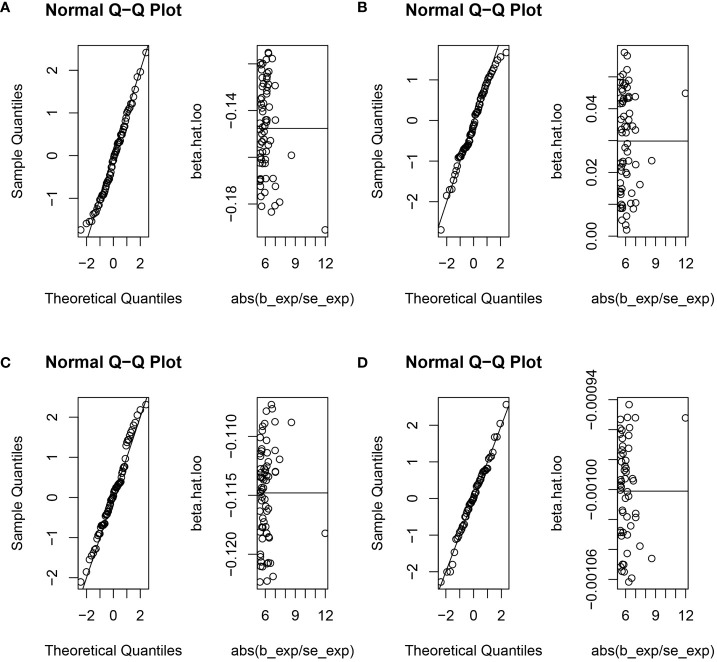
The normal distribution between age at first birth and four common esophageal diseases. **(A)** age at first birth and oesophageal obstruction; **(B)** age at first birth and oesophageal varices; **(C)** age at first birth and gastro-oesophageal reflux; **(D)** age at first birth and oesophageal cancer.

Cochran’s Q statistic, employed in the context of MR with the MR-IVW method, and Rucker’s Q statistic, utilized in the framework of MR Egger, both demonstrated a lack of heterogeneity in the genetic causal assessment between AFB and four distinct outcomes (OO, OV, GOR, OC), as presented in **Table 1**. Furthermore, the results of the intercept tests conducted within the MR Egger and MR-PRESSO analyses consistently indicated the absence of horizontal pleiotropy in the assessment of genetic causality between AFB and the four aforementioned outcomes, as detailed in **Table 1**. Notably, the MR-PRESSO analysis did not reveal any outliers in the genetic causal assessment of AFB and the four outcomes, as illustrated in **Table 1**. Moreover, the “Leave-one-out” analysis, as depicted in **Figure 4**, demonstrated that the genetic causal assessment between AFB and the four outcomes (OO, OV, GOR, OC) remained robust and unaffected by the exclusion of any SNP. Lastly, the MR-RAPS analysis, visually represented in **Figure 5**, indicated that the genetic causal assessment between AFB and the four outcomes (OO, OV, GOR, OC) adhered to a normal distribution pattern.

**Table 1:** Sensitivity analysis of the MR analysis results of exposure and outcome.

Exposure	Outcome	Heterogeneity		Pleiotropy	MR-PRESSO		MR-RAPS
		Cochran’s Q Test (IVW)	Rucker’s Q Test (MR-Egger)	Intercept Test (MR-Egger)	Outliers	Pleiotropy	Normal Distribution
		P value	P value	P value	Numbers	P value	P value
Age at first birth	Oesophageal obstruction	0.292	0.261	0.912	0	0.477	0.299
	Oesophageal varices	0.840	0.815	0.877	0	0.787	0.475
	Gastro-oesophageal reflux	0.327	0.296	0.776	0	0.142	0.386
	Oesophageal cancer	0.211	0.183	0.954	0	0.400	0.921

**Figure 6:**
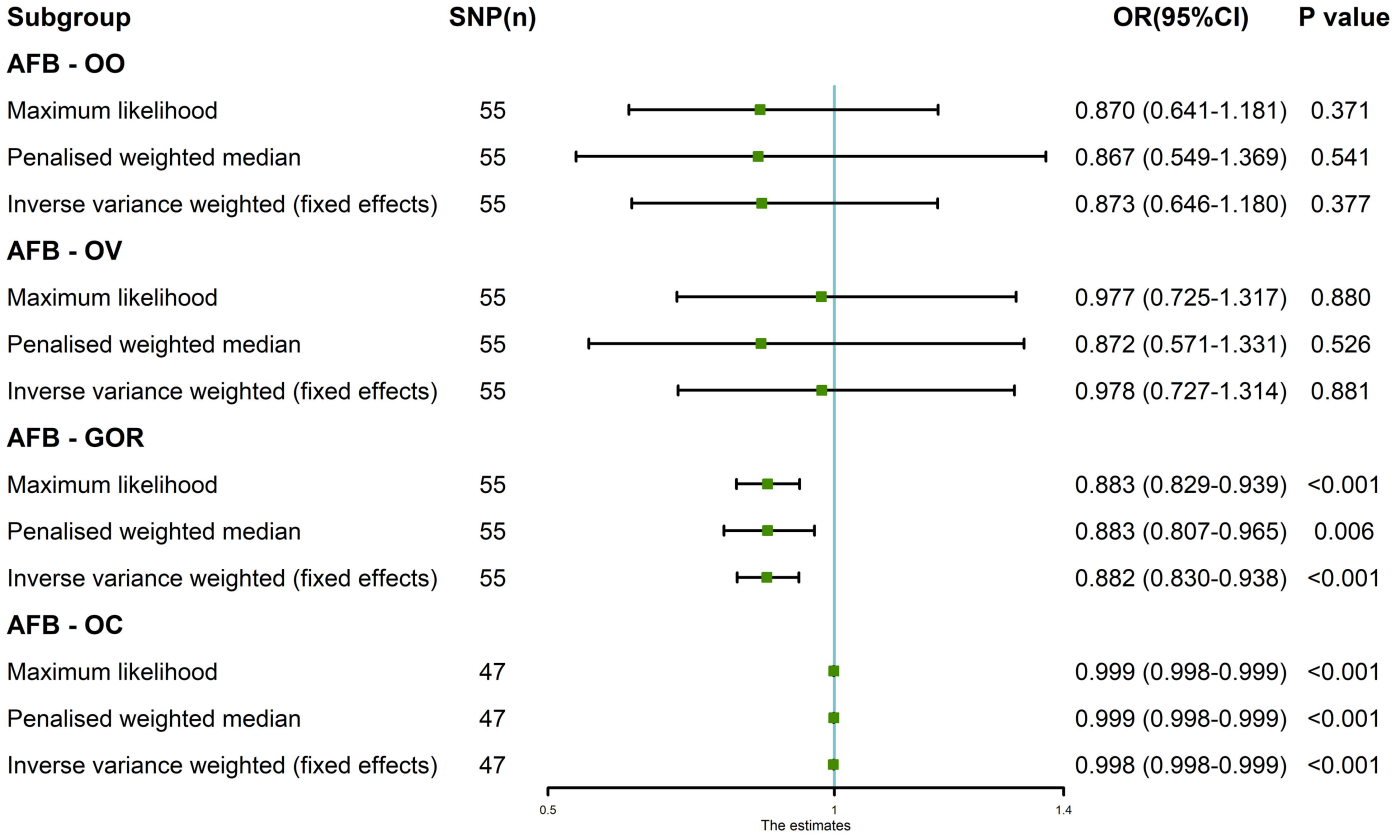
The MR analysis results of age at first birth and four common esophageal diseases, including oesophageal obstruction, oesophageal varices, gastro-oesophageal reflux and oesophageal cancer. The analysis employed three methods, namely maximum likelihood, penalized weighted median, and fixed-effects IVW.

Finally, the genetic causality assessment between AFB and four distinct outcomes (OO, OV, GOR, OC) was subjected to validation through the application of three distinct validation methods, namely, maximum likelihood estimation, penalized weighted median estimation, and fixed-effects IVW estimation. The analytical outcomes were found to be concordant with those obtained using the random-effects IVW. The findings of this investigation revealed a lack of genetic causality between AFB and both OO and OV (P > 0.05), while indicating a negative genetic causality association between AFB and both GOR and OC (P < 0.05, OR < 1) as visually depicted in **Figure 6**.

## 4 Discussion

This research endeavor sought to explore the genetic causal connection between AFB and a quartet of esophageal disorders, namely, OO, OV, GOR, and OC, employing MR analysis as the investigative method. Our genetic causal investigation uncovered a negative genetic causal association between AFB and the occurrence of GOR and OC, implying that a younger AFB is associated with an elevated risk of GOR and OC, while delayed childbearing may act as a protective factor against the onset of GOR and OC. Furthermore, our genetic causal appraisal indicates the absence of a genetic-level causal linkage between AFB and OO or OV. However, the pathogenesis of the disease is complex, and although AFB and OO or OV are not causally related at the genetic level, it cannot be ruled out that they have some relationship at other levels than genetics.

AFB is recognized as a reliable metric for assessing intricate reproductive outcomes and is frequently employed as a pivotal parameter for forecasting demographic trends. The evidence substantiates that genetic factors contribute substantially, potentially accounting for up to 50% of variations in reproductive behaviors, including AFB and the number of children ever borne (NEB) (29). The study found that AFB exhibits positive genetic correlations with the age of menarche, the occurrence of a broken voice, and educational attainment. Conversely, the presence of a higher number of alleles associated with an increase in AFB is associated with a reduced genetic predisposition to smoking, obesity, and diabetes (30). This underscores the substantial genetic underpinnings of AFB and its close nexus with human health and developmental trajectories.

Prior genetic causal investigations have established a significant relationship between AFB factors in advanced age and their causal role in mitigating the progression of lung cancer (19). Moreover, heightened AFB has been causally associated with a decreased risk of postpartum depression (31). The findings of the present study elucidate that, at a genetic level, an elevation in AFB is linked to a reduced risk of GOR with an OR of 0.882, whereas an increase in AFB confers a relatively weak protective effect against OC with an OR of 0.998. We think that this phenomenon may be correlated with the presence of precancerous lesions, with GOR representing a precursor to OC, and the upsurge in AFB serves to prevent the onset of GOR, consequently indirectly safeguarding against the incidence of OC. As a result, the direct protective influence of AFB elevation on OC is observed to be less pronounced compared to its effect on GOR. Prior investigations have yielded conflicting outcomes concerning the association between AFB and OC. A case-control study conducted within a Swedish population indicated that childbirth reduced the risk of OC among women compared to those who had not given birth, with AFB exhibiting no significant effect on women but a discernible impact on men (32). Conversely, a case-control study within a Chinese population suggested that delayed childbearing may heighten the risk of OC in women (33). Several epidemiological inquiries have explored the role of hormonal and reproductive factors in OC risk development. A study utilizing the UK Biobank cohort revealed an inverse correlation between older age at the first and final live birth and OC, while stillbirths, miscarriages, and terminations were positively associated with OC (34). An observational meta-analysis focused on reproductive factors and OC risk disclosed that age at menopause and hormone replacement therapy were linked to reduced OC risk, whereas postmenopausal status was associated with an increased OC risk (6). A cohort study encompassing a large female population demonstrated that women who had not given birth exhibited a higher risk of OC compared to their counterparts who had experienced childbirth (35). Other reproductive factors, namely age at menarche, age at menopause, genital removal surgery, and breastfeeding, have also been investigation regarding their potential associations with OC risk, although a majority of these variables did not exhibit statistically significant relationships (36–38).

Estrogen signaling plays a pivotal role in modulating adipose tissue metabolism, potentially establishing a correlation between estrogen levels and male obesity, a notable predisposing factor for OC (9). Given the marked sex-specific disparities in OC incidence, it is plausible to infer the involvement of the estrogen signaling network in OC pathogenesis. Furthermore, considering the higher occurrence of OC in men, the significance of androgens in this context becomes evident. The expression of the androgen receptor (AR) in OC is noteworthy, as studies have indicated a reduction in the occurrence of esophageal squamous carcinoma (ESCC) and esophageal adenocarcinoma (EAC) subsequent to androgen deprivation therapy (39). Investigations have reported a diminished risk of OC associated with menopausal hormone therapy in women (40). As women age, there is a decline in their physical function, a decline more closely associated with reproductive age than chronological age. Notably, this decline in physical function does not appear to be solely attributed to alterations in three reproductive hormones—anti-mullerian hormone (AMH), follicle-stimulating hormone (FSH), and luteinizing hormone (LH)—during menopausal transition (41). Moreover, studies have observed an association between gene-predicted FSH and LH levels and the risk of EAC (42). Through this analysis, it becomes apparent that the correlation between reproductive hormones and OC may operate on a genetic framework, contrasting with the link between reproductive age and female physiological capability, which appears to remain distinct from the fluctuations in reproductive hormones such as AMH, FSH, and LH. Therefore, it is reasonable to postulate that the association between AFB, hormones, and OC may also operate at the genetic level. This aligns with the genetic-level findings of this study, supporting AFB as a potential protective factor against OC.

In this investigation, we examined the genetic causal relationship between AFB and four prevalent esophageal conditions, through MR analysis. MR analysis is robust against confounding variables and reverse causality, offering a degree of reliability. Nevertheless, akin to prior MR analyses, this study is not without its constraints. Firstly, the study cohort consisted of European participants, thereby warranting prudence in generalizing our findings to other populations. Secondly, for the analysis of OC, gender stratification should be carried out, which may have more clinical significance and research value. Despite evident sex differences in the context of OC, the current limitations of GWAS data impede the opportunity to distinguish between sexes for genetic causal evaluation. It is believed that that as the purview of GWAS research continues to expand, future research will hopefully solve this problem.

## 5 Conclusion

This study employs MR analysis to investigate the genetic causal links between AFB and four distinct esophageal disorders. The findings unveil a noteworthy negative genetic causation pattern between AFB and GOR and OC. These results suggest that a younger AFB might constitute a risk factor for GOR and OC, while conversely, delaying childbirth may confer protective benefits against these conditions. Moreover, our investigation does not yield substantiating evidence for a genetic causal association between AFB and either OO or OV. The outcomes of this study have practical implications, shedding light on the relevance of AFB in clinical contexts, particularly emphasizing the significance of early childbearing age with regard to GOR and OC incidence.

## Data availability statement

This study utilized publicly available datasets, which wereobtained from the IEU OpenGWAS database (https://gwas.mrcieu.ac.uk/) and FinnGen consortium (https://www.finngen.fi/). 

## Author contributions

YS: Data curation, Methodology, Writing – original draft. YX: Data curation, Methodology, Writing – review & editing. YH: Investigation, Methodology, Writing – review & editing. YC: Software, Writing – review & editing. FW: Software, Writing – review & editing. MY: Conceptualization, Data curation, Writing – review & editing. YP: Conceptualization, Funding acquisition, Supervision, Writing – review & editing.
