# Supplementary material for: Late age at first birth is a protective factor for oesophageal cancer and gastro-oesophageal reflux: the evidence from the genetic study
Source: Front Endocrinol (Lausanne). 2024 Jan 15;14:1329763. doi: 10.3389/fendo.2023.1329763 (PMC10823002; doi:10.3389/fendo.2023.1329763)
Supplement: Supplementary file 1 [file Table_1.doc]

**Supplementary Table 1:** The data used in this study.

| **GWAS ID** | **Year** | **Trait** | **Samples** | **SNPs** | **Population** |
| --- | --- | --- | --- | --- | --- |
| ebi-a-GCST90000050 | 2021 | Age at first birth | 542,901 | 9,702,772 | European |
| finn-b-K11_OESOBST | 2021 | Oesophageal obstruction | 190,156 | 16,380,373 | European |
| finn-b-I9_VARICVEOES | 2021 | Oesophageal varices | 190,513 | 16,380,395 | European |
| finn-b-K11_REFLUX | 2021 | Gastro-oesophageal reflux | 202,836 | 16,380,425 | European |
| ieu-b-4960 | 2021 | Oesophageal cancer | 372,756 | 8,970,465 | European |

**Supplementary Table 2:** The instrumental variables used in MR analysis between age at first birth and oesophageal obstruction.

|  | **SNP** | **beta.exposure** | **se.exposure** | **pval.exposure** | **pval.outcome** | **palindromic** | **F** |
| --- | --- | --- | --- | --- | --- | --- | --- |
| 1 | rs10445366 | -0.0886 | 0.0136 | 6.69E-11 | 0.8669 | TRUE | 42.44124 |
| 2 | rs10752613 | -0.0715 | 0.0102 | 1.89E-12 | 0.7851 | TRUE | 49.13717 |
| 3 | rs10786831 | 0.0519 | 0.0092 | 1.68E-08 | 0.8326 | FALSE | 31.8242 |
| 4 | rs10941692 | 0.0702 | 0.0114 | 8.84E-10 | 0.784 | FALSE | 37.91953 |
| 5 | rs10962552 | -0.0707 | 0.0128 | 3.12E-08 | 0.6204 | FALSE | 30.50825 |
| 6 | rs11081529 | -0.0595 | 0.0099 | 1.81E-09 | 0.611399 | FALSE | 36.12118 |
| 7 | rs11167753 | -0.0574 | 0.01 | 1.04E-08 | 0.8928 | FALSE | 32.94748 |
| 8 | rs112282597 | 0.0631 | 0.0112 | 1.57E-08 | 0.4807 | FALSE | 31.74103 |
| 9 | rs11242222 | 0.0686 | 0.0122 | 1.67E-08 | 0.054 | FALSE | 31.61746 |
| 10 | rs11249939 | 0.0633 | 0.0099 | 1.56E-10 | 0.5634 | FALSE | 40.88231 |
| 11 | rs112512729 | 0.0965 | 0.0175 | 3.50E-08 | 0.07657 | FALSE | 30.40723 |
| 12 | rs113247159 | 0.0641 | 0.0115 | 2.68E-08 | 0.1225 | FALSE | 31.06839 |
| 13 | rs11774212 | 0.0551 | 0.0096 | 1.08E-08 | 0.078291 | FALSE | 32.9427 |
| 14 | rs11887646 | 0.0707 | 0.0112 | 2.25E-10 | 0.3027 | FALSE | 39.84751 |
| 15 | rs11915934 | -0.073 | 0.0122 | 2.25E-09 | 0.5276 | FALSE | 35.80342 |
| 16 | rs12407439 | 0.0754 | 0.0138 | 4.37E-08 | 0.6151 | FALSE | 29.85265 |
| 17 | rs12815613 | 0.0564 | 0.0097 | 5.75E-09 | 0.276 | FALSE | 33.80751 |
| 18 | rs13319205 | -0.0646 | 0.0106 | 9.85E-10 | 0.726501 | TRUE | 37.14084 |
| 19 | rs13413037 | -0.0577 | 0.0097 | 2.30E-09 | 0.1987 | FALSE | 35.38397 |
| 20 | rs13420733 | 0.0595 | 0.0104 | 1.22E-08 | 0.6842 | TRUE | 32.73148 |
| 21 | rs13420733 | 0.0595 | 0.0104 | 1.22E-08 | 0.1823 | TRUE | 32.73148 |
| 22 | rs142800469 | 0.0562 | 0.0099 | 1.31E-08 | 0.5963 | FALSE | 32.22557 |
| 23 | rs1430901 | 0.0562 | 0.0093 | 1.33E-09 | 0.3444 | FALSE | 36.51784 |
| 24 | rs1464534 | 0.0606 | 0.0099 | 1.00E-09 | 0.8156 | TRUE | 37.4691 |
| 25 | rs1590949 | 0.0583 | 0.0095 | 8.24E-10 | 0.4238 | TRUE | 37.66069 |
| 26 | rs1606974 | -0.0747 | 0.0137 | 4.61E-08 | 0.1377 | FALSE | 29.73024 |
| 27 | rs1702877 | 0.0602 | 0.0098 | 8.22E-10 | 0.2492 | FALSE | 37.73455 |
| 28 | rs17314804 | 0.0662 | 0.0095 | 2.67E-12 | 0.1329 | FALSE | 48.55871 |
| 29 | rs17391694 | -0.083 | 0.0135 | 8.15E-10 | 0.4103 | FALSE | 37.79959 |
| 30 | rs17731405 | -0.0735 | 0.0129 | 1.21E-08 | 0.2051 | FALSE | 32.46337 |
| 31 | rs1859100 | -0.0539 | 0.0094 | 1.08E-08 | 0.647099 | FALSE | 32.87912 |
| 32 | rs2069278 | -0.0576 | 0.0097 | 3.13E-09 | 0.8742 | FALSE | 35.26143 |
| 33 | rs2253763 | -0.0534 | 0.0098 | 4.58E-08 | 0.8319 | FALSE | 29.69127 |
| 34 | rs2347867 | 0.0818 | 0.0095 | 5.04E-18 | 0.8418 | FALSE | 74.14089 |
| 35 | rs2530597 | -0.0549 | 0.0097 | 1.70E-08 | 0.9893 | TRUE | 32.03315 |
| 36 | rs2906457 | 0.0649 | 0.0103 | 2.92E-10 | 0.8517 | FALSE | 39.70209 |
| 37 | rs293566 | -0.0694 | 0.0099 | 2.18E-12 | 0.408 | FALSE | 49.14134 |
| 38 | rs33920398 | -0.0573 | 0.0104 | 3.93E-08 | 0.2694 | FALSE | 30.35575 |
| 39 | rs34137317 | 0.2473 | 0.04 | 6.15E-10 | 0.3713 | FALSE | 38.22317 |
| 40 | rs359271 | -0.0622 | 0.0098 | 1.88E-10 | 0.2104 | FALSE | 40.28348 |
| 41 | rs4244533 | -0.0719 | 0.0119 | 1.33E-09 | 0.7802 | FALSE | 36.50583 |
| 42 | rs4443016 | 0.0706 | 0.0095 | 1.02E-13 | 0.2545 | TRUE | 55.22816 |
| 43 | rs55988458 | 0.0726 | 0.0119 | 1.16E-09 | 0.1445 | FALSE | 37.22012 |
| 44 | rs57432042 | 0.0905 | 0.0165 | 4.57E-08 | 0.6402 | FALSE | 30.08345 |
| 45 | rs5763436 | -0.0542 | 0.0097 | 1.94E-08 | 0.566301 | TRUE | 31.22148 |
| 46 | rs590076 | -0.0553 | 0.0095 | 5.78E-09 | 0.5254 | FALSE | 33.88453 |
| 47 | rs60222682 | -0.0646 | 0.0117 | 3.51E-08 | 0.3346 | FALSE | 30.48539 |
| 48 | rs61750814 | -0.0796 | 0.0129 | 6.32E-10 | 0.2148 | FALSE | 38.07546 |
| 49 | rs62261746 | 0.0576 | 0.0104 | 2.85E-08 | 0.4766 | TRUE | 30.67444 |
| 50 | rs6574018 | 0.0556 | 0.01 | 2.42E-08 | 0.01442 | FALSE | 30.91349 |
| 51 | rs6585429 | 0.084 | 0.0124 | 1.08E-11 | 0.4869 | FALSE | 45.88953 |
| 52 | rs6677536 | 0.0553 | 0.0101 | 3.85E-08 | 0.8276 | FALSE | 29.97823 |
| 53 | rs6908444 | -0.0558 | 0.0102 | 4.51E-08 | 0.9311 | FALSE | 29.92723 |
| 54 | rs6923535 | -0.052 | 0.0093 | 2.12E-08 | 0.5261 | FALSE | 31.26361 |
| 55 | rs693691 | -0.077 | 0.0135 | 1.26E-08 | 0.3074 | FALSE | 32.53212 |
| 56 | rs72704712 | -0.0879 | 0.0133 | 4.56E-11 | 0.2036 | FALSE | 43.67902 |
| 57 | rs72779695 | -0.0849 | 0.0145 | 4.87E-09 | 0.8523 | FALSE | 34.28292 |
| 58 | rs7516843 | 0.0611 | 0.0097 | 3.12E-10 | 0.04683 | FALSE | 39.67687 |
| 59 | rs78928669 | 0.142 | 0.0259 | 4.22E-08 | 0.3042 | FALSE | 30.05907 |
| 60 | rs7958796 | -0.0676 | 0.0101 | 2.15E-11 | 0.1487 | TRUE | 44.79701 |
| 61 | rs80153284 | 0.2314 | 0.0411 | 1.74E-08 | 0.1927 | FALSE | 31.6987 |
| 62 | rs8030494 | 0.0545 | 0.0096 | 1.30E-08 | 0.8672 | FALSE | 32.22916 |
| 63 | rs8110682 | 0.0695 | 0.011 | 2.59E-10 | 0.07028 | FALSE | 39.91927 |
| 64 | rs9401333 | -0.0705 | 0.0101 | 3.24E-12 | 0.3845 | FALSE | 48.72298 |
| 65 | rs9540715 | 0.0588 | 0.01 | 4.74E-09 | 0.9536 | FALSE | 34.57427 |
| 66 | rs9818010 | 0.063 | 0.0102 | 5.63E-10 | 0.2209 | FALSE | 38.14865 |
| 67 | rs9838987 | -0.1175 | 0.0098 | 5.55E-33 | 0.4674 | FALSE | 143.7547 |

**Supplementary Table 3:** The instrumental variables used in MR analysis between age at first birth and oesophageal varices.

|  | **SNP** | **beta.exposure** | **se.exposure** | **pval.exposure** | **pval.outcome** | **palindromic** | **F** |
| --- | --- | --- | --- | --- | --- | --- | --- |
| 1 | rs10445366 | -0.0886 | 0.0136 | 6.69E-11 | 0.1361 | TRUE | 42.44124 |
| 2 | rs10752613 | -0.0715 | 0.0102 | 1.89E-12 | 0.3898 | TRUE | 49.13717 |
| 3 | rs10786831 | 0.0519 | 0.0092 | 1.68E-08 | 0.8997 | FALSE | 31.8242 |
| 4 | rs10941692 | 0.0702 | 0.0114 | 8.84E-10 | 0.068151 | FALSE | 37.91953 |
| 5 | rs10962552 | -0.0707 | 0.0128 | 3.12E-08 | 0.174 | FALSE | 30.50825 |
| 6 | rs11081529 | -0.0595 | 0.0099 | 1.81E-09 | 0.08314 | FALSE | 36.12118 |
| 7 | rs11167753 | -0.0574 | 0.01 | 1.04E-08 | 0.2063 | FALSE | 32.94748 |
| 8 | rs112282597 | 0.0631 | 0.0112 | 1.57E-08 | 0.4385 | FALSE | 31.74103 |
| 9 | rs11242222 | 0.0686 | 0.0122 | 1.67E-08 | 0.4621 | FALSE | 31.61746 |
| 10 | rs11249939 | 0.0633 | 0.0099 | 1.56E-10 | 0.6243 | FALSE | 40.88231 |
| 11 | rs112512729 | 0.0965 | 0.0175 | 3.50E-08 | 0.2651 | FALSE | 30.40723 |
| 12 | rs113247159 | 0.0641 | 0.0115 | 2.68E-08 | 0.2936 | FALSE | 31.06839 |
| 13 | rs11774212 | 0.0551 | 0.0096 | 1.08E-08 | 0.5437 | FALSE | 32.9427 |
| 14 | rs11887646 | 0.0707 | 0.0112 | 2.25E-10 | 0.7558 | FALSE | 39.84751 |
| 15 | rs11915934 | -0.073 | 0.0122 | 2.25E-09 | 0.2952 | FALSE | 35.80342 |
| 16 | rs12407439 | 0.0754 | 0.0138 | 4.37E-08 | 0.3856 | FALSE | 29.85265 |
| 17 | rs12815613 | 0.0564 | 0.0097 | 5.75E-09 | 0.5039 | FALSE | 33.80751 |
| 18 | rs13319205 | -0.0646 | 0.0106 | 9.85E-10 | 0.006571 | TRUE | 37.14084 |
| 19 | rs13413037 | -0.0577 | 0.0097 | 2.30E-09 | 0.9015 | FALSE | 35.38397 |
| 20 | rs13420733 | 0.0595 | 0.0104 | 1.22E-08 | 0.2716 | TRUE | 32.73148 |
| 21 | rs13420733 | 0.0595 | 0.0104 | 1.22E-08 | 0.5128 | TRUE | 32.73148 |
| 22 | rs142800469 | 0.0562 | 0.0099 | 1.31E-08 | 0.2143 | FALSE | 32.22557 |
| 23 | rs1430901 | 0.0562 | 0.0093 | 1.33E-09 | 0.9219 | FALSE | 36.51784 |
| 24 | rs1464534 | 0.0606 | 0.0099 | 1.00E-09 | 0.5715 | TRUE | 37.4691 |
| 25 | rs1590949 | 0.0583 | 0.0095 | 8.24E-10 | 0.8585 | TRUE | 37.66069 |
| 26 | rs1606974 | -0.0747 | 0.0137 | 4.61E-08 | 0.4403 | FALSE | 29.73024 |
| 27 | rs1702877 | 0.0602 | 0.0098 | 8.22E-10 | 0.2254 | FALSE | 37.73455 |
| 28 | rs17314804 | 0.0662 | 0.0095 | 2.67E-12 | 0.5652 | FALSE | 48.55871 |
| 29 | rs17391694 | -0.083 | 0.0135 | 8.15E-10 | 0.4045 | FALSE | 37.79959 |
| 30 | rs17731405 | -0.0735 | 0.0129 | 1.21E-08 | 0.3796 | FALSE | 32.46337 |
| 31 | rs1859100 | -0.0539 | 0.0094 | 1.08E-08 | 0.7267 | FALSE | 32.87912 |
| 32 | rs2069278 | -0.0576 | 0.0097 | 3.13E-09 | 0.8481 | FALSE | 35.26143 |
| 33 | rs2253763 | -0.0534 | 0.0098 | 4.58E-08 | 0.3473 | FALSE | 29.69127 |
| 34 | rs2347867 | 0.0818 | 0.0095 | 5.04E-18 | 0.808 | FALSE | 74.14089 |
| 35 | rs2530597 | -0.0549 | 0.0097 | 1.70E-08 | 0.5371 | TRUE | 32.03315 |
| 36 | rs2906457 | 0.0649 | 0.0103 | 2.92E-10 | 0.523701 | FALSE | 39.70209 |
| 37 | rs293566 | -0.0694 | 0.0099 | 2.18E-12 | 0.7515 | FALSE | 49.14134 |
| 38 | rs33920398 | -0.0573 | 0.0104 | 3.93E-08 | 0.697 | FALSE | 30.35575 |
| 39 | rs34137317 | 0.2473 | 0.04 | 6.15E-10 | 0.4671 | FALSE | 38.22317 |
| 40 | rs359271 | -0.0622 | 0.0098 | 1.88E-10 | 0.561 | FALSE | 40.28348 |
| 41 | rs4244533 | -0.0719 | 0.0119 | 1.33E-09 | 0.5489 | FALSE | 36.50583 |
| 42 | rs4443016 | 0.0706 | 0.0095 | 1.02E-13 | 0.5757 | TRUE | 55.22816 |
| 43 | rs55988458 | 0.0726 | 0.0119 | 1.16E-09 | 0.1569 | FALSE | 37.22012 |
| 44 | rs57432042 | 0.0905 | 0.0165 | 4.57E-08 | 0.090301 | FALSE | 30.08345 |
| 45 | rs5763436 | -0.0542 | 0.0097 | 1.94E-08 | 0.596901 | TRUE | 31.22148 |
| 46 | rs590076 | -0.0553 | 0.0095 | 5.78E-09 | 0.7086 | FALSE | 33.88453 |
| 47 | rs60222682 | -0.0646 | 0.0117 | 3.51E-08 | 0.361 | FALSE | 30.48539 |
| 48 | rs61750814 | -0.0796 | 0.0129 | 6.32E-10 | 0.9473 | FALSE | 38.07546 |
| 49 | rs62261746 | 0.0576 | 0.0104 | 2.85E-08 | 0.7546 | TRUE | 30.67444 |
| 50 | rs6574018 | 0.0556 | 0.01 | 2.42E-08 | 0.6694 | FALSE | 30.91349 |
| 51 | rs6585429 | 0.084 | 0.0124 | 1.08E-11 | 0.3319 | FALSE | 45.88953 |
| 52 | rs6677536 | 0.0553 | 0.0101 | 3.85E-08 | 0.4941 | FALSE | 29.97823 |
| 53 | rs6908444 | -0.0558 | 0.0102 | 4.51E-08 | 0.4844 | FALSE | 29.92723 |
| 54 | rs6923535 | -0.052 | 0.0093 | 2.12E-08 | 0.8665 | FALSE | 31.26361 |
| 55 | rs693691 | -0.077 | 0.0135 | 1.26E-08 | 0.5002 | FALSE | 32.53212 |
| 56 | rs72704712 | -0.0879 | 0.0133 | 4.56E-11 | 0.5177 | FALSE | 43.67902 |
| 57 | rs72779695 | -0.0849 | 0.0145 | 4.87E-09 | 0.1417 | FALSE | 34.28292 |
| 58 | rs7516843 | 0.0611 | 0.0097 | 3.12E-10 | 0.763199 | FALSE | 39.67687 |
| 59 | rs78928669 | 0.142 | 0.0259 | 4.22E-08 | 0.5298 | FALSE | 30.05907 |
| 60 | rs7958796 | -0.0676 | 0.0101 | 2.15E-11 | 0.8781 | TRUE | 44.79701 |
| 61 | rs80153284 | 0.2314 | 0.0411 | 1.74E-08 | 0.1116 | FALSE | 31.6987 |
| 62 | rs8030494 | 0.0545 | 0.0096 | 1.30E-08 | 0.2366 | FALSE | 32.22916 |
| 63 | rs8110682 | 0.0695 | 0.011 | 2.59E-10 | 0.3864 | FALSE | 39.91927 |
| 64 | rs9401333 | -0.0705 | 0.0101 | 3.24E-12 | 0.9125 | FALSE | 48.72298 |
| 65 | rs9540715 | 0.0588 | 0.01 | 4.74E-09 | 0.0959 | FALSE | 34.57427 |
| 66 | rs9818010 | 0.063 | 0.0102 | 5.63E-10 | 0.8604 | FALSE | 38.14865 |
| 67 | rs9838987 | -0.1175 | 0.0098 | 5.55E-33 | 0.7824 | FALSE | 143.7547 |

**Supplementary Table 4:** The instrumental variables used in MR analysis between age at first birth and gastro-oesophageal reflux.

|  | **SNP** | **beta.exposure** | **se.exposure** | **pval.exposure** | **pval.outcome** | **palindromic** | **F** |
| --- | --- | --- | --- | --- | --- | --- | --- |
| 1 | rs10445366 | -0.0886 | 0.0136 | 6.69E-11 | 0.073629 | TRUE | 42.44124 |
| 2 | rs10752613 | -0.0715 | 0.0102 | 1.89E-12 | 0.3938 | TRUE | 49.13717 |
| 3 | rs10786831 | 0.0519 | 0.0092 | 1.68E-08 | 0.1456 | FALSE | 31.8242 |
| 4 | rs10941692 | 0.0702 | 0.0114 | 8.84E-10 | 0.9324 | FALSE | 37.91953 |
| 5 | rs10962552 | -0.0707 | 0.0128 | 3.12E-08 | 0.2587 | FALSE | 30.50825 |
| 6 | rs11081529 | -0.0595 | 0.0099 | 1.81E-09 | 0.3891 | FALSE | 36.12118 |
| 7 | rs11167753 | -0.0574 | 0.01 | 1.04E-08 | 0.748101 | FALSE | 32.94748 |
| 8 | rs112282597 | 0.0631 | 0.0112 | 1.57E-08 | 0.7782 | FALSE | 31.74103 |
| 9 | rs11242222 | 0.0686 | 0.0122 | 1.67E-08 | 0.2239 | FALSE | 31.61746 |
| 10 | rs11249939 | 0.0633 | 0.0099 | 1.56E-10 | 0.528 | FALSE | 40.88231 |
| 11 | rs112512729 | 0.0965 | 0.0175 | 3.50E-08 | 0.1962 | FALSE | 30.40723 |
| 12 | rs113247159 | 0.0641 | 0.0115 | 2.68E-08 | 0.4254 | FALSE | 31.06839 |
| 13 | rs11774212 | 0.0551 | 0.0096 | 1.08E-08 | 0.9269 | FALSE | 32.9427 |
| 14 | rs11887646 | 0.0707 | 0.0112 | 2.25E-10 | 0.9036 | FALSE | 39.84751 |
| 15 | rs11915934 | -0.073 | 0.0122 | 2.25E-09 | 0.4364 | FALSE | 35.80342 |
| 16 | rs12407439 | 0.0754 | 0.0138 | 4.37E-08 | 0.1514 | FALSE | 29.85265 |
| 17 | rs12815613 | 0.0564 | 0.0097 | 5.75E-09 | 0.6217 | FALSE | 33.80751 |
| 18 | rs13319205 | -0.0646 | 0.0106 | 9.85E-10 | 0.5208 | TRUE | 37.14084 |
| 19 | rs13413037 | -0.0577 | 0.0097 | 2.30E-09 | 0.757201 | FALSE | 35.38397 |
| 20 | rs13420733 | 0.0595 | 0.0104 | 1.22E-08 | 0.491801 | TRUE | 32.73148 |
| 21 | rs13420733 | 0.0595 | 0.0104 | 1.22E-08 | 0.1094 | TRUE | 32.73148 |
| 22 | rs142800469 | 0.0562 | 0.0099 | 1.31E-08 | 0.757501 | FALSE | 32.22557 |
| 23 | rs1430901 | 0.0562 | 0.0093 | 1.33E-09 | 0.869 | FALSE | 36.51784 |
| 24 | rs1464534 | 0.0606 | 0.0099 | 1.00E-09 | 0.1132 | TRUE | 37.4691 |
| 25 | rs1590949 | 0.0583 | 0.0095 | 8.24E-10 | 0.748101 | TRUE | 37.66069 |
| 26 | rs1606974 | -0.0747 | 0.0137 | 4.61E-08 | 0.928 | FALSE | 29.73024 |
| 27 | rs1702877 | 0.0602 | 0.0098 | 8.22E-10 | 0.4505 | FALSE | 37.73455 |
| 28 | rs17314804 | 0.0662 | 0.0095 | 2.67E-12 | 0.05023 | FALSE | 48.55871 |
| 29 | rs17391694 | -0.083 | 0.0135 | 8.15E-10 | 0.3725 | FALSE | 37.79959 |
| 30 | rs17731405 | -0.0735 | 0.0129 | 1.21E-08 | 0.050751 | FALSE | 32.46337 |
| 31 | rs1859100 | -0.0539 | 0.0094 | 1.08E-08 | 0.006097 | FALSE | 32.87912 |
| 32 | rs2069278 | -0.0576 | 0.0097 | 3.13E-09 | 0.00345 | FALSE | 35.26143 |
| 33 | rs2253763 | -0.0534 | 0.0098 | 4.58E-08 | 0.2016 | FALSE | 29.69127 |
| 34 | rs2347867 | 0.0818 | 0.0095 | 5.04E-18 | 0.09391 | FALSE | 74.14089 |
| 35 | rs2530597 | -0.0549 | 0.0097 | 1.70E-08 | 0.7474 | TRUE | 32.03315 |
| 36 | rs2906457 | 0.0649 | 0.0103 | 2.92E-10 | 0.3562 | FALSE | 39.70209 |
| 37 | rs293566 | -0.0694 | 0.0099 | 2.18E-12 | 0.3821 | FALSE | 49.14134 |
| 38 | rs33920398 | -0.0573 | 0.0104 | 3.93E-08 | 0.3182 | FALSE | 30.35575 |
| 39 | rs34137317 | 0.2473 | 0.04 | 6.15E-10 | 0.2663 | FALSE | 38.22317 |
| 40 | rs359271 | -0.0622 | 0.0098 | 1.88E-10 | 0.7552 | FALSE | 40.28348 |
| 41 | rs4244533 | -0.0719 | 0.0119 | 1.33E-09 | 0.7391 | FALSE | 36.50583 |
| 42 | rs4443016 | 0.0706 | 0.0095 | 1.02E-13 | 0.282 | TRUE | 55.22816 |
| 43 | rs55988458 | 0.0726 | 0.0119 | 1.16E-09 | 0.009488 | FALSE | 37.22012 |
| 44 | rs57432042 | 0.0905 | 0.0165 | 4.57E-08 | 0.07033 | FALSE | 30.08345 |
| 45 | rs5763436 | -0.0542 | 0.0097 | 1.94E-08 | 0.2003 | TRUE | 31.22148 |
| 46 | rs590076 | -0.0553 | 0.0095 | 5.78E-09 | 0.5111 | FALSE | 33.88453 |
| 47 | rs60222682 | -0.0646 | 0.0117 | 3.51E-08 | 0.3902 | FALSE | 30.48539 |
| 48 | rs61750814 | -0.0796 | 0.0129 | 6.32E-10 | 0.278 | FALSE | 38.07546 |
| 49 | rs62261746 | 0.0576 | 0.0104 | 2.85E-08 | 0.2146 | TRUE | 30.67444 |
| 50 | rs6574018 | 0.0556 | 0.01 | 2.42E-08 | 0.667599 | FALSE | 30.91349 |
| 51 | rs6585429 | 0.084 | 0.0124 | 1.08E-11 | 0.3567 | FALSE | 45.88953 |
| 52 | rs6677536 | 0.0553 | 0.0101 | 3.85E-08 | 0.6885 | FALSE | 29.97823 |
| 53 | rs6908444 | -0.0558 | 0.0102 | 4.51E-08 | 0.6877 | FALSE | 29.92723 |
| 54 | rs6923535 | -0.052 | 0.0093 | 2.12E-08 | 0.4769 | FALSE | 31.26361 |
| 55 | rs693691 | -0.077 | 0.0135 | 1.26E-08 | 0.5987 | FALSE | 32.53212 |
| 56 | rs72704712 | -0.0879 | 0.0133 | 4.56E-11 | 0.02872 | FALSE | 43.67902 |
| 57 | rs72779695 | -0.0849 | 0.0145 | 4.87E-09 | 0.7061 | FALSE | 34.28292 |
| 58 | rs7516843 | 0.0611 | 0.0097 | 3.12E-10 | 0.2988 | FALSE | 39.67687 |
| 59 | rs78928669 | 0.142 | 0.0259 | 4.22E-08 | 0.4438 | FALSE | 30.05907 |
| 60 | rs7958796 | -0.0676 | 0.0101 | 2.15E-11 | 0.01403 | TRUE | 44.79701 |
| 61 | rs80153284 | 0.2314 | 0.0411 | 1.74E-08 | 0.6272 | FALSE | 31.6987 |
| 62 | rs8030494 | 0.0545 | 0.0096 | 1.30E-08 | 0.6476 | FALSE | 32.22916 |
| 63 | rs8110682 | 0.0695 | 0.011 | 2.59E-10 | 0.4565 | FALSE | 39.91927 |
| 64 | rs9401333 | -0.0705 | 0.0101 | 3.24E-12 | 0.2189 | FALSE | 48.72298 |
| 65 | rs9540715 | 0.0588 | 0.01 | 4.74E-09 | 0.3656 | FALSE | 34.57427 |
| 66 | rs9818010 | 0.063 | 0.0102 | 5.63E-10 | 0.03932 | FALSE | 38.14865 |
| 67 | rs9838987 | -0.1175 | 0.0098 | 5.55E-33 | 0.5499 | FALSE | 143.7547 |

**Supplementary Table 5:** The instrumental variables used in MR analysis between age at first birth and oesophageal cancer.

|  | **SNP** | **beta.exposure** | **se.exposure** | **pval.exposure** | **pval.outcome** | **palindromic** | **F** |
| --- | --- | --- | --- | --- | --- | --- | --- |
| 1 | rs10445366 | -0.0886 | 0.0136 | 6.69E-11 | 0.88 | TRUE | 42.44124 |
| 2 | rs10752613 | -0.0715 | 0.0102 | 1.89E-12 | 0.83 | TRUE | 49.13717 |
| 3 | rs10786831 | 0.0519 | 0.0092 | 1.68E-08 | 0.21 | FALSE | 31.8242 |
| 4 | rs10941692 | 0.0702 | 0.0114 | 8.84E-10 | 0.56 | FALSE | 37.91953 |
| 5 | rs10962552 | -0.0707 | 0.0128 | 3.12E-08 | 0.26 | FALSE | 30.50825 |
| 6 | rs11081529 | -0.0595 | 0.0099 | 1.81E-09 | 0.52 | FALSE | 36.12118 |
| 7 | rs11167753 | -0.0574 | 0.01 | 1.04E-08 | 0.24 | FALSE | 32.94748 |
| 8 | rs112282597 | 0.0631 | 0.0112 | 1.57E-08 | 0.12 | FALSE | 31.74103 |
| 9 | rs11242222 | 0.0686 | 0.0122 | 1.67E-08 | 0.099001 | FALSE | 31.61746 |
| 10 | rs11249939 | 0.0633 | 0.0099 | 1.56E-10 | 0.1 | FALSE | 40.88231 |
| 11 | rs112512729 | 0.0965 | 0.0175 | 3.50E-08 | 0.52 | FALSE | 30.40723 |
| 12 | rs113247159 | 0.0641 | 0.0115 | 2.68E-08 | 0.51 | FALSE | 31.06839 |
| 13 | rs11774212 | 0.0551 | 0.0096 | 1.08E-08 | 0.82 | FALSE | 32.9427 |
| 14 | rs11887646 | 0.0707 | 0.0112 | 2.25E-10 | 0.14 | FALSE | 39.84751 |
| 15 | rs11915934 | -0.073 | 0.0122 | 2.25E-09 | 0.69 | FALSE | 35.80342 |
| 16 | rs12407439 | 0.0754 | 0.0138 | 4.37E-08 | 0.55 | FALSE | 29.85265 |
| 17 | rs12815613 | 0.0564 | 0.0097 | 5.75E-09 | 0.23 | FALSE | 33.80751 |
| 18 | rs13319205 | -0.0646 | 0.0106 | 9.85E-10 | 0.39 | TRUE | 37.14084 |
| 19 | rs13413037 | -0.0577 | 0.0097 | 2.30E-09 | 0.47 | FALSE | 35.38397 |
| 20 | rs13420733 | 0.0595 | 0.0104 | 1.22E-08 | 0.86 | TRUE | 32.73148 |
| 21 | rs142800469 | 0.0562 | 0.0099 | 1.31E-08 | 0.14 | FALSE | 32.22557 |
| 22 | rs1430901 | 0.0562 | 0.0093 | 1.33E-09 | 0.4 | FALSE | 36.51784 |
| 23 | rs1464534 | 0.0606 | 0.0099 | 1.00E-09 | 0.32 | TRUE | 37.4691 |
| 24 | rs1590949 | 0.0583 | 0.0095 | 8.24E-10 | 0.92 | TRUE | 37.66069 |
| 25 | rs1606974 | -0.0747 | 0.0137 | 4.61E-08 | 0.1 | FALSE | 29.73024 |
| 26 | rs1702877 | 0.0602 | 0.0098 | 8.22E-10 | 0.23 | FALSE | 37.73455 |
| 27 | rs17314804 | 0.0662 | 0.0095 | 2.67E-12 | 0.99 | FALSE | 48.55871 |
| 28 | rs17391694 | -0.083 | 0.0135 | 8.15E-10 | 0.46 | FALSE | 37.79959 |
| 29 | rs17731405 | -0.0735 | 0.0129 | 1.21E-08 | 0.13 | FALSE | 32.46337 |
| 30 | rs1859100 | -0.0539 | 0.0094 | 1.08E-08 | 0.023 | FALSE | 32.87912 |
| 31 | rs2069278 | -0.0576 | 0.0097 | 3.13E-09 | 0.35 | FALSE | 35.26143 |
| 32 | rs2253763 | -0.0534 | 0.0098 | 4.58E-08 | 0.075999 | FALSE | 29.69127 |
| 33 | rs2347867 | 0.0818 | 0.0095 | 5.04E-18 | 0.95 | FALSE | 74.14089 |
| 34 | rs2530597 | -0.0549 | 0.0097 | 1.70E-08 | 0.29 | TRUE | 32.03315 |
| 35 | rs2906457 | 0.0649 | 0.0103 | 2.92E-10 | 0.66 | FALSE | 39.70209 |
| 36 | rs293566 | -0.0694 | 0.0099 | 2.18E-12 | 0.77 | FALSE | 49.14134 |
| 37 | rs33920398 | -0.0573 | 0.0104 | 3.93E-08 | 0.13 | FALSE | 30.35575 |
| 38 | rs34137317 | 0.2473 | 0.04 | 6.15E-10 | 0.083 | FALSE | 38.22317 |
| 39 | rs359271 | -0.0622 | 0.0098 | 1.88E-10 | 0.0014 | FALSE | 40.28348 |
| 40 | rs4244533 | -0.0719 | 0.0119 | 1.33E-09 | 0.37 | FALSE | 36.50583 |
| 41 | rs4443016 | 0.0706 | 0.0095 | 1.02E-13 | 0.93 | TRUE | 55.22816 |
| 42 | rs55988458 | 0.0726 | 0.0119 | 1.16E-09 | 0.11 | FALSE | 37.22012 |
| 43 | rs57432042 | 0.0905 | 0.0165 | 4.57E-08 | 0.0053 | FALSE | 30.08345 |
| 44 | rs5763436 | -0.0542 | 0.0097 | 1.94E-08 | 0.74 | TRUE | 31.22148 |
| 45 | rs590076 | -0.0553 | 0.0095 | 5.78E-09 | 0.43 | FALSE | 33.88453 |
| 46 | rs61750814 | -0.0796 | 0.0129 | 6.32E-10 | 0.85 | FALSE | 38.07546 |
| 47 | rs62261746 | 0.0576 | 0.0104 | 2.85E-08 | 0.51 | TRUE | 30.67444 |
| 48 | rs6574018 | 0.0556 | 0.01 | 2.42E-08 | 0.59 | FALSE | 30.91349 |
| 49 | rs6585429 | 0.084 | 0.0124 | 1.08E-11 | 0.46 | FALSE | 45.88953 |
| 50 | rs6677536 | 0.0553 | 0.0101 | 3.85E-08 | 0.8 | FALSE | 29.97823 |
| 51 | rs6908444 | -0.0558 | 0.0102 | 4.51E-08 | 0.62 | FALSE | 29.92723 |
| 52 | rs6923535 | -0.052 | 0.0093 | 2.12E-08 | 0.31 | FALSE | 31.26361 |
| 53 | rs693691 | -0.077 | 0.0135 | 1.26E-08 | 0.19 | FALSE | 32.53212 |
| 54 | rs72704712 | -0.0879 | 0.0133 | 4.56E-11 | 0.4 | FALSE | 43.67902 |
| 55 | rs72779695 | -0.0849 | 0.0145 | 4.87E-09 | 0.2 | FALSE | 34.28292 |
| 56 | rs7359501 | 0.0565 | 0.0094 | 1.82E-09 | 0.3 | FALSE | 36.12764 |
| 57 | rs7516843 | 0.0611 | 0.0097 | 3.12E-10 | 0.21 | FALSE | 39.67687 |
| 58 | rs78928669 | 0.142 | 0.0259 | 4.22E-08 | 0.82 | FALSE | 30.05907 |
| 59 | rs7958796 | -0.0676 | 0.0101 | 2.15E-11 | 0.44 | TRUE | 44.79701 |
| 60 | rs8030494 | 0.0545 | 0.0096 | 1.30E-08 | 0.88 | FALSE | 32.22916 |
| 61 | rs8110682 | 0.0695 | 0.011 | 2.59E-10 | 0.29 | FALSE | 39.91927 |
| 62 | rs9401333 | -0.0705 | 0.0101 | 3.24E-12 | 0.079001 | FALSE | 48.72298 |
| 63 | rs9540715 | 0.0588 | 0.01 | 4.74E-09 | 0.38 | FALSE | 34.57427 |
| 64 | rs9818010 | 0.063 | 0.0102 | 5.63E-10 | 0.61 | FALSE | 38.14865 |
| 65 | rs9838987 | -0.1175 | 0.0098 | 5.55E-33 | 0.097 | FALSE | 143.7547 |
| Confounders | |  | | | | | |
| 1 | rs11081529 | Past tobacco smoking | | | | | |
| 2 | rs13319205 | Ever smoked | | | | | |
| 3 | rs1464534 | Ever smoked | | | | | |
| 4 | rs1702877 | Alcohol intake frequency | | | | | |
| 5 | rs17314804 | Ever smoked | | | | | |
| 6 | rs17391694 | Alcohol intake frequency | | | | | |
| 7 | rs1859100 | Current tobacco smoking | | | | | |
| 8 | rs2530597 | Alcohol intake frequency | | | | | |
| 9 | rs55988458 | Alcohol intake frequency | | | | | |
| 10 | rs590076 | Current tobacco smoking | | | | | |
| 11 | rs62261746 | Ever smoked | | | | | |
